# Mapping the Potential Global Codling Moth (*Cydia pomonella L*.) Distribution Based on a Machine Learning Method

**DOI:** 10.1038/s41598-018-31478-3

**Published:** 2018-08-30

**Authors:** Dong Jiang, Shuai Chen, Mengmeng Hao, Jingying Fu, Fangyu Ding

**Affiliations:** 1 State Key Laboratory of Resources and Environmental Information Systems, Institute of Geographical Sciences and Natural Resources Research, Chinese Academy of Sciences, Beijing, 100101 China; 2 College of Resource and Environment, University of Chinese Academy of Sciences, Beijing, 100049 China; 3 Key Laboratory of Carrying Capacity Assessment for Resource and Environment, Ministry of Land & Resources, Beijing, 100101 China

## Abstract

The spread of invasive species may pose great threats to the economy and ecology of a region. The codling moth (*Cydia pomonella L*.) is one of the 100 worst invasive alien species in the world and is the most destructive apple pest. The economic losses caused by codling moths are immeasurable. It is essential to understand the potential distribution of codling moths to reduce the risks of codling moth establishment. In this study, we adopted the Maxent (Maximum Entropy Model), a machine learning method to predict the potential global distribution of codling moths with global accessibility data, apple yield data, elevation data and 19 bioclimatic variables, considering the ecological characteristics and the spread channels that cover the processes from growth and survival to the dispersion of the codling moth. The results show that the areas that are suitable for codling moth are mainly distributed in Europe, Asia and North America, and these results strongly conformed with the currently known occurrence regions. In addition, global accessibility, mean temperature of the coldest quarter, precipitation of the driest month, annual mean temperature and apple yield were the most important environmental predictors associated with the global distribution of codling moths.

## Introduction

Biological invasions could result in serious global consequences if not handled properly, including ecological destruction and economic losses. Especially in agriculture, crop losses and pest control can be extremely expensive^1^. The increases in international trade and transportation have established novel pathways for the spread of invasive species^2^, which worsens the situation.

The codling moth, *Cydia pomonella L*. (Lepidoptera: Tortricide) is one of the most detrimental and economically important apple pests, and the moth has the potential to cause complete crop losses in untreated apple orchards^3^. The codling moth is a multivoltine species, and adaptive behaviour, such as facultative diapause and multiple generations per breeding season, have allowed the codling moth to adapt to diverse climatic conditions. Although the flight capacity of the codling moth is limited^4^, they can spread over long distances through the transportation of infested fruit and packing material, and this has become the most common method for colonization of new habitats. The codling moth is considered to have originated from south-eastern Europe^5^, over the last two centuries, they have dispersed throughout the world and have reached almost global distribution. The codling moth is now a cosmopolitan insect that occurs in almost every country where apples are grown, becoming one of the most successful pest insect species in terms of invasiveness^6^.

Prevention of biological invasions is much less expensive than post-entry control^1^. Detailed knowledge of the geographic and ecological distribution of a species is fundamental for conservation planning and forecasting^7^. To reduce the ecological destruction and economic losses caused by codling moth invasions, it is essential to understand the potential distribution of codling moths for risk assessment and decision making. Ecological niche models (ENM) have become an effective tool for assessing the potential risk for establishment of invasive species in recent years. There are basically two types of ecological niche models used: correlative models (e.g., Maxent, GARP, ENFA) and process-based distribution models (e.g.; CLIMEX). These models were used to estimate the potential risk of codling moths by some researchers. Liang *et al*. analysed the suitability for codling moths in China based on biological data of codling moths and meteorological data from 760 weather sites by using CLIMEX and ArcGIS^8^. Svobodova *et al*. investigated the historical occurrence of the codling moth in southern Moravia and northern Austria by using CLIMEX^9^. Vavrovic *et al*. used the CLIMEX model to estimate the potential codling moth infestation pressure in Slovakia under the conditions of climate change^10^. Zhao *et al*. adopted an ecological niche model, Maxent, to interpret the disjunct distribution and potential distribution of codling moths in China and identified the relative roles of climate, humans and vegetation with respect to the present codling moth distribution^11^. Kumar *et al*. used CLIMEX and Maxent model to map the global risk of codling moth establishment and compared the results of the two models^12^.

However, there are some limitations of the current studies: Firstly, the existing codling moth occurrence records that have been used to fit the models are inadequate. Secondly, most of the studies have merely focused on climate conditions for the establishment of codling moths, ignoring the availability of host plants and the increased possibility of transportation. Although climatic conditions are a major determinant of the potential distribution of codling moths, the risk of establishment of codling moths in new places is also largely influenced by human factors. Thirdly, many studies have adopted mechanism models, which are suitable for macroscopical predictions, but the performance at local scales is not good. Lastly, most research is based on regional studies, and there are few studies on the potential distribution of codling moths at the global scale. To solve these problems, in this study, codling moth occurrence records were collected from multiple sources. In addition, a maximum entropy model, which is a machine learning method, was used to simulate the potential global distribution of codling moths with global accessibility data, apple yield data, elevation data and 19 bioclimatic variables, considering the ecological characteristics and the expansion channels that cover the processes from growth and survival to the dispersal of codling moths.

## Results

### Potential distribution of codling moth

**Figure 1:**
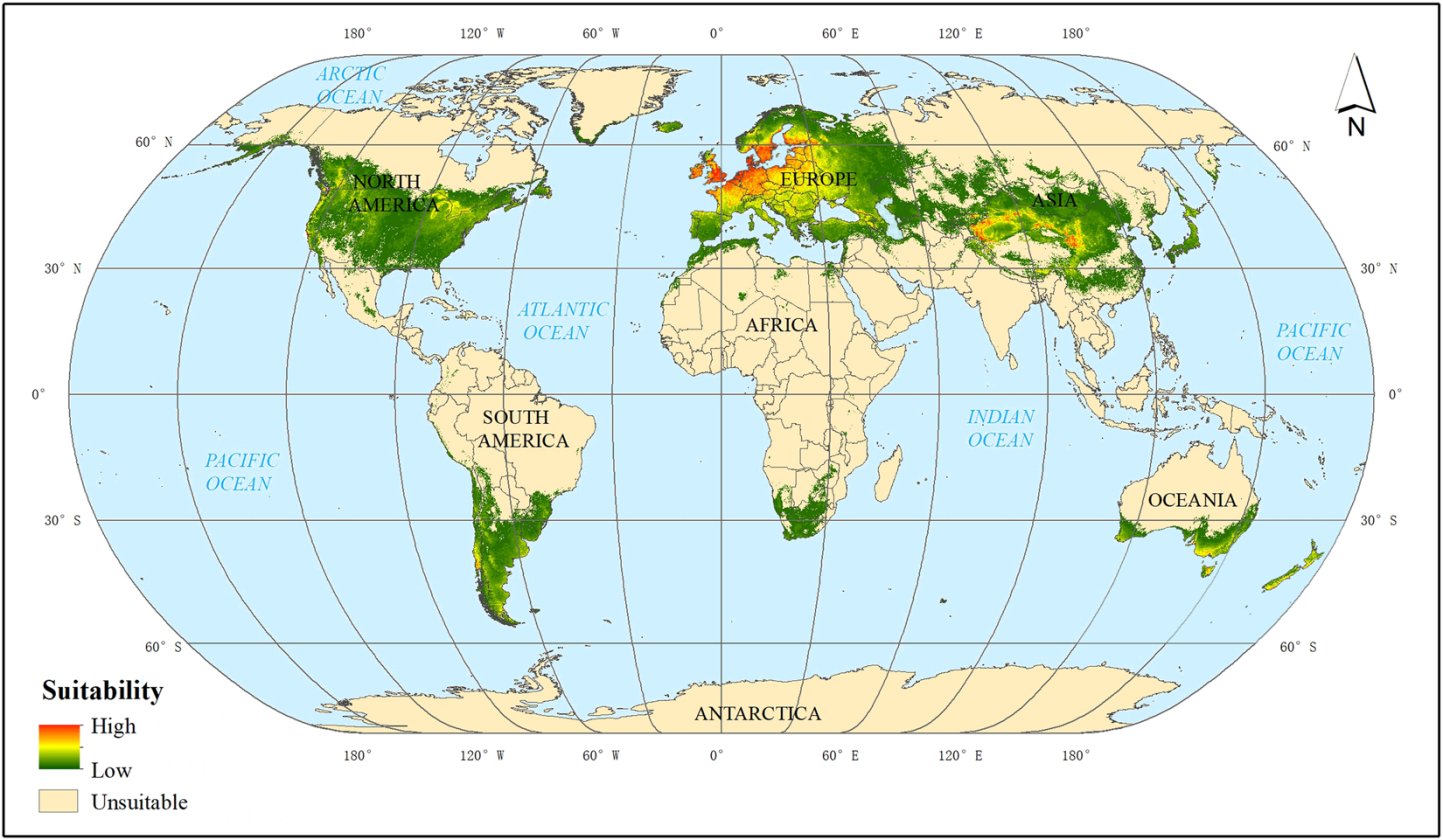
Global potential distribution of codling moth using Maxent.

The potential distribution of codling moth predicted by the Maxent model is in good agreement with the current known codling moth occurrence regions (Fig. 1). Overall, the areas that were predicted to be suitable for codling moths are distributed on all continents except Antarctica. The suitable regions for codling moth are mainly distributed in Europe, North America and Asia. It is noteworthy that few areas between the latitudes of 20°N and 20°S or beyond 70°N and 70°S are predicted to be suitable for codling moths. By contrast, most of the suitable areas are distributed between the latitudes of 30° and 60°. In addition, the distribution of codling moths is significantly different on different continents.

In Asia (Supplementary Fig. S1), the areas that were predicted to be suitable for codling moths are primarily distributed in Central Asia and East Asia. The model predicted higher codling moth suitability in China but lower suitability in Central Asian countries including Kazakhstan, Uzbekistan, and Tajikistan. The suitable regions covered most of the apple-growing countries such as China, Turkey, Azerbaijan, Japan, North Korea, South Korea, India, Iran, Pakistan and Kazakhstan. In China, the model predicted no or very low suitability in the southern provinces and Qinghai-Tibetan Plateau, which might be the result of the lack of host plants. Medium or highly suitable areas are distributed in most of the apple-producing provinces including Xinjiang, Gansu, Shanxi, Shaanxi, Shandong, Hebei, and Liaoning. In addition, it should be noted that the model predicted suitable conditions for codling moths in some regions where codling moths do not yet occur, such as South Korea and Japan, but there are host plants in these regions that are preferred by codling moths such as apple trees.

The areas that were predicted to be suitable almost cover all of Europe (Supplementary Fig. S2), which closely matches the known codling moth European distribution. This area also seems to have the highest predicted suitability for codling moth. On the one hand, codling moths are believed to have originated from somewhere in south-eastern Europe where there are favourable climate conditions for growth and reproduction. On the other hand, there are sufficient occurrence records in Europe, and this could be another reason for the high suitability. The suitable areas covered almost all apple-growing European countries including Poland, Italy, France, the Netherlands, Belgium, Spain, Austria, Germany, Russian Federation, Hungary, Portugal, the United Kingdom, and Switzerland. The model predicted highly suitable areas in the United Kingdom, Germany, France, Poland, Belgium, the Netherlands, Denmark and the southern parts of Sweden and Finland, which is consistent with the current known distribution of codling moth.

In North America (Supplementary Fig. S3), the Maxent model predicted medium suitability in the eastern United States and relatively high suitability on the west coast. The model also predicted suitable conditions in the southern parts of Alaska and Greenland. The model predicted low suitability in the southeast and southwest corners of Canada and the central part of Mexico. The three countries of North America are all apple-growing and exporting countries.

In South America (Supplementary Fig. S4), the areas that were predicted to be suitable for codling moth by the Maxent model were mainly distributed along the Andes mountain range and in the southern parts of the continent, including the Patagonia Plateau, Pampas grasslands and Parana Plateau. The suitability of these areas is relatively low, covering the major apple-growing countries in North America such as Chile, Argentina, Brazil and Peru. The model predicted no or little suitable area in low-latitude regions including most of the Central American countries.

In Oceania (Supplementary Fig. S5), the suitable codling moth areas that were predicted by the Maxent model were mainly distributed in south and south-eastern Australia and New Zealand. Both Australia and New Zealand are apple-growing countries. The predicted suitability for codling moth is relatively high in the south-east of Australia, and the suitability gradually declines from the coast to inland areas.

The fewest suitable areas were in Africa (Supplementary Fig. S6), and the Maxent model predicted only a few small areas with suitable environmental conditions for codling moths. These areas are mainly located in the southern and northern coastal regions such as South Africa, Morocco and Algeria, and all three of these countries are major apple-growing countries in Africa. It is remarkable that there are no occurrence records in either Africa or North America, but the Maxent model predicted suitable areas on both continents, and these areas are consistent with the current apple-planting areas. This result indicates that the environments in these areas are appropriate for the growth and reproduction of codling moth, and also demonstrates the predictive power of the Maxent model.

### Accuracy analysis

**Figure 2:**
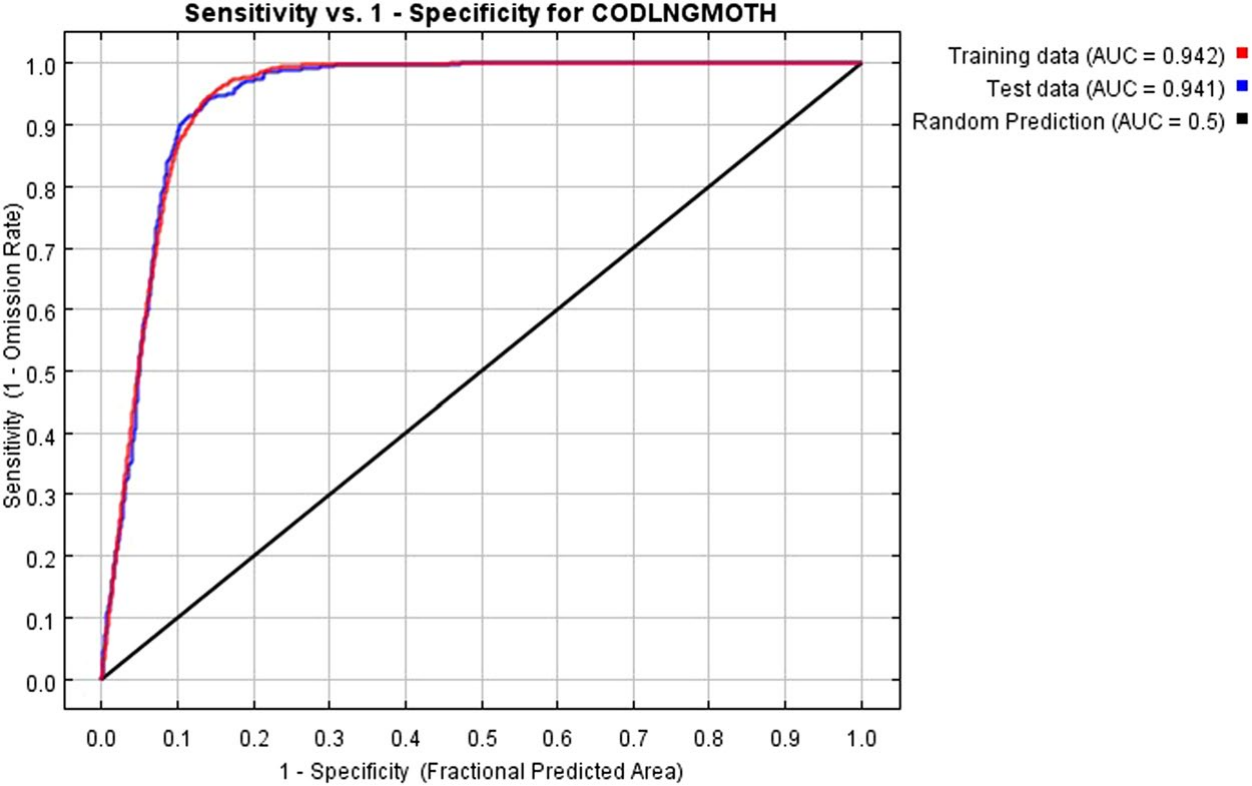
Receiver operating characteristic (ROC) curve for the Maxent model, the AUC (area under the receiver operating characteristic curve) values vary from 0 to 1; values < 0.5 indicate that the model performance is worse than random, 0.5 indicates performance that is not better than random, 0.5–0.7 indicates poor performance, 0.7–0.9 indicates reasonable or moderate performance, and >0.9 indicates high performance^34^.

**Figure 3:**
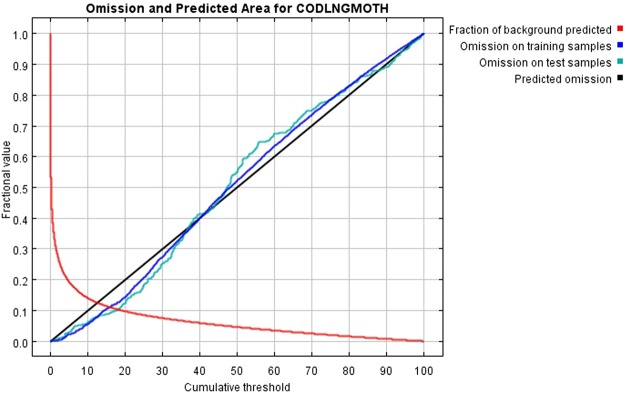
Omission and predicted areas for codling moth; lower omission rates represent better model performance.

By using 22 factor layers and 1776 presence locations (25% was set aside for testing) as the input data for Maxent, the model results show that this model performed well, and the AUC values of the training data and test data were 0.942 and 0.941, respectively (Fig. 2), which strongly supports its predictive power. In addition, the model had low omission rates (Fig. 3). A low omission rate is a necessary (but not sufficient) condition for a good model^13^.

### Effects of environmental factors

**Figure 4:**
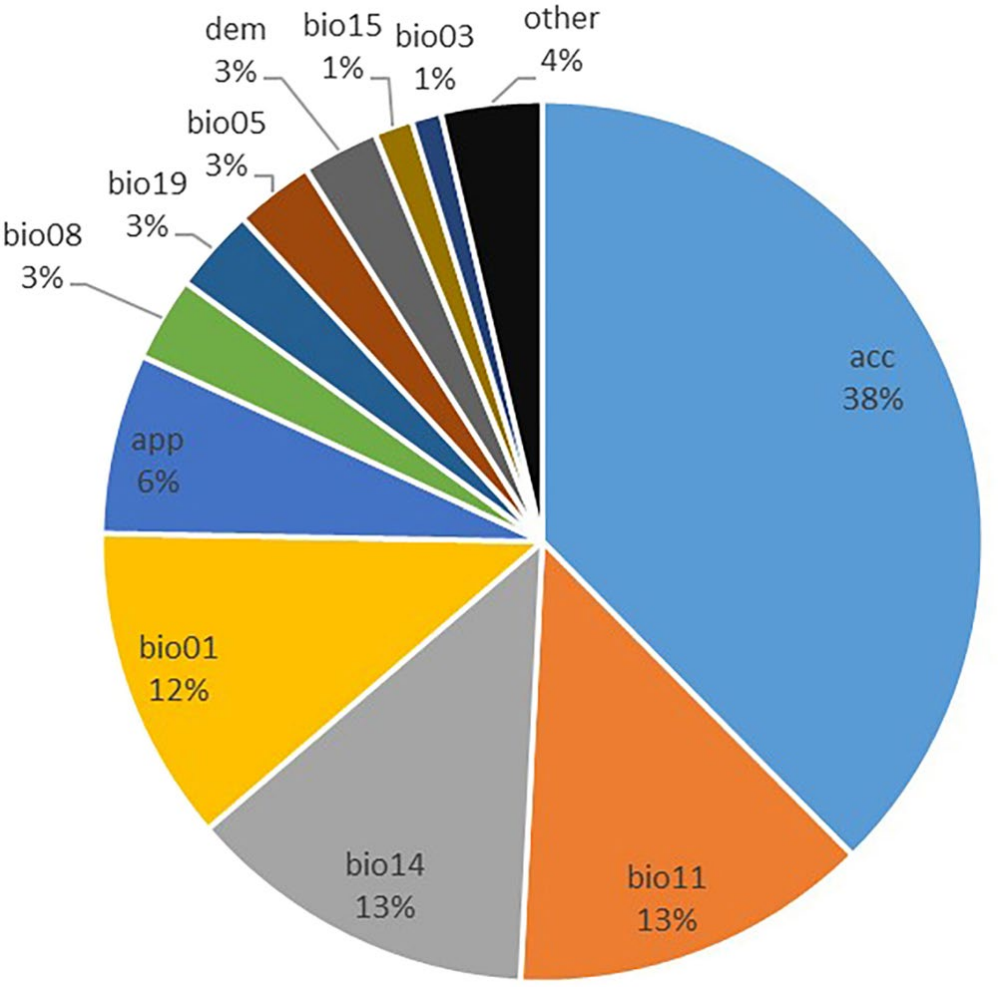
Relative contributions of the environmental variables to the model.

The following chart (Fig. 4) gives estimates of the relative contributions of each variable to the distribution of codling moth. From the statistics, global accessibility, mean temperature of the coldest quarter, precipitation of the driest month, annual mean temperature and apple yield were the most important environmental predictors associated with codling moth global distribution with average contributions to the Maxent model of 38%, 13%, 13%, 12%, and 6%, respectively.

From the contribution of each variable, we can see that global accessibility and apple yield have great impacts on the potential distribution of codling moths in addition to climatic conditions. Therefore, it is necessary to consider these factors when predicting codling moth invasions.

## Discussion

In the present study, we adopted the Maxent machine learning method to assess the potential global distribution risk of codling moths. Codling moth occurrence records were collected from multisource as many as possible. In addition to climatic variables, the availability of host plants together with international trade and transportation were also taken into account, covering the processes from growth and survival to the dispersion of codling moth. The AUC values indicate that the model performed well, and a global suitability map for codling moth was generated.

By comparison with other studies, the overall codling moth distribution range is roughly the same, but our study included a better representation of details. Results from different studies agreed with each other in major codling moth occurrence regions, for example, in Europe, North America, central Chile, Argentina, northern Morocco, northern Algeria, South Africa, southern Australia, New Zealand, Central Asia and East Asia. The predicted areas with high suitability in all three studies are mainly distributed in Europe, Asia and North America, which conformed to the current known distribution of codling moth.

However, the environmental suitability of codling moth in this study was significantly different from Zhao’s^11^ and Kumar’s^12^. In the study of Kumar, it predicted more suitable areas in Central Asia and northeast China than this study. By contrast, our study predicted more suitable areas in Northern Europe, such as Norway, Sweden and Finland. One of the reasons might be the different distribution of occurrence records, another is that we considered more input variables such as global accessibility and apple yield data. Besides, our study has different suitability distribution patterns comparing with Kumar and Zhao. In Kumar’s study, the degree of suitability was distributed roughly along latitude lines, suitability in two hemispheres was almost symmetrical regardless of the discontinuous continents. In our study, the predicted suitability for codling moth is less regularly distributed, looks similar to Zhao’s study from the global scale. But there are notable differences from a local perspective, the suitability in our study included more details that highly correlated with the global accessibility.

The model also predicted suitable environments for codling moths in some regions where they do not occur yet but include their favourite host plants. Finally, the major codling moth predictors were extracted, and global accessibility, mean temperature of the coldest quarter, precipitation of the driest month, annual mean temperature and apple yield were the most important predictors associated with the global distribution of codling moths. All of this information is very useful in assessing the risk of codling moth colonization in new areas.

However, this study has some defects that can be ameliorated by additional research. The codling moth occurrence data are still insufficient in some regions, and the Maxent model prediction may be affected by occurrence points that are not uniformly distributed. In addition, biological invasions are very complex processes. There are some other factors that affect the potential distribution of codling moths. Therefore, more complex models and more elements will be our next research directions.

## Methods

The main work of this paper can be summarized as the following aspects.

Step 1: Codling moth occurrence records were collected from multiple sources, ensuring the highest possible data integrity.

Step 2: A high-resolution spatial dataset was produced, which included the ecological characteristics and the expansion channels that cover the processes from growth and survival to the invasion of codling moth.

Step 3: A Maxent model was built to simulate the potential global distribution of codling moths.

**Figure 5:**
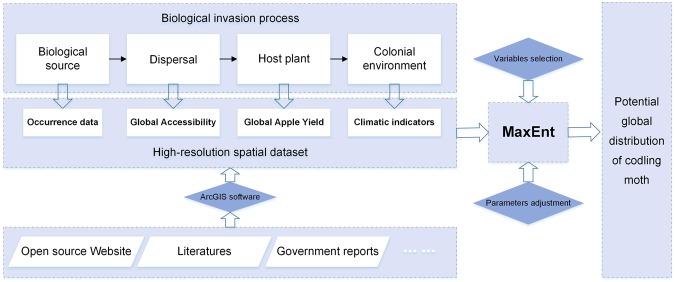
Technical flow chart of this study.

The technical flow chart of this study is shown in Fig. 5.

### Occurrence data

**Figure 6:**
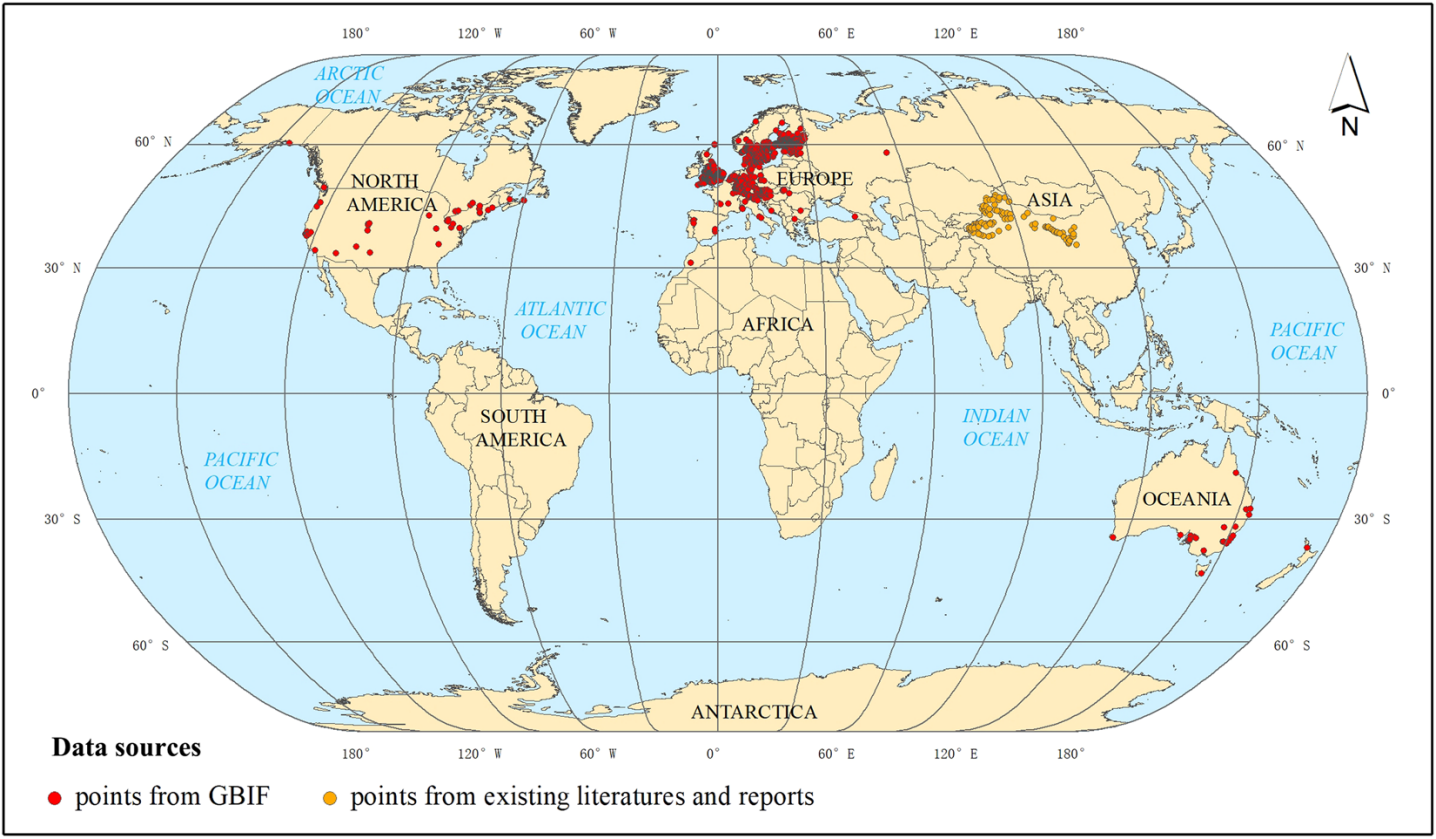
Worldwide codling moth occurrence records. The map was generated by ArcGIS 10.2 software^35^; the red points represent the occurrence records from the GBIF, and the yellow points represent the occurrence records extracted from the existing literature and reports.

Georeferenced occurrence data of codling moths were collected from three different sources: (1) Existing open source data, which were mainly accessed via the online Global Biodiversity Information Facility database^14^, which is one of the most popular species distribution data sources. (2) Published articles and maps of codling moth occurrences were also used to extract occurrence location information. (3) Government documents, reports and related supportive materials were also used. Occurrence data from the GBIF covered most regions in the world where the codling moth is known to occur except Asia, South America and Africa. Therefore, occurrence data from published literatures and government reports were used as supplemental material for the GBIF data. After removing duplicate occurrence records, a total of 1776 occurrence records were collected for Maxent input occurrence data (Fig. 6).

### Factor data

The factor data needed for the Maxent model consists of bioclimatic variables, global accessibility, apple yield data and elevation data (Table 1). The bioclimatic variable data were acquired from the World-Clim dataset^15^ with a resolution of 30 arc seconds, which is approximately 1 km^2^; these bioclimatic variables are more biologically meaningful variables that were derived from the monthly temperature and rainfall values, representing annual trends (e.g., mean annual temperature, annual precipitation), seasonality (e.g., annual ranges of temperature and precipitation) and extreme or limiting environmental factors (e.g., temperature of the coldest and warmest months and precipitation of the wet and dry quarters). The global accessibility map was download from the Joint Research Centre of the European Commission’s science and knowledge service^16^, and it reflects the global connectivity of transportation and the concentration of economic activities; the pixel values on the map represent the travel time in minutes to major cities with a resolution of 30 arc seconds, which is approximately 1 km^2^. The apple production data were acquired from EarthStat^17^ with a 5-minute spatial resolution, which is approximately 10 km^2^; these data represent the total apple production in metric tons on the land-area mass of a grid cell. The elevation data were obtained from the NASA Shuttle Radar Topography Mission (SRTM)^18^, which provides high-quality digital elevation models (DEM) for the entire globe with a spatial resolution of 3 arc seconds, which is approximately 250 m. To ensure spatial consistency of these variables, we converted the spatial resolutions of all data to 0.05 degrees.

**Table 1:** Variables for Maxent input.

Categories	Variables	Description	Data Source
dispersal means	acc	global accessibility	JRC of European Commission
availability of host plants	app	global apple production	EarthStat
climatic indicators	dem	global elevation	SRTM
	bio1	annual mean temperature	World Clim version 2
	bio2	mean diurnal range	
	bio3	isothermality (bio2/bio7) (* 100)	
	bio4	temperature seasonality	
	bio5	max temperature of warmest month	
	bio6	min temperature of coldest month	
	bio7	temperature annual range (bio5-bio6)	
	bio8	mean temperature of wettest quarter	
	bio9	mean temperature of driest quarter	
	bio10	mean temperature of warmest quarter	
	bio11	mean temperature of coldest quarter	
	bio12	annual precipitation	
	bio13	precipitation of wettest month	
	bio14	precipitation of driest month	
	bio15	precipitation seasonality	
	bio16	precipitation of wettest quarter	
	bio17	precipitation of driest quarter	
	bio18	precipitation of warmest quarter	
	bio19	precipitation of coldest quarter	

The 22 variables were selected as Maxent model inputs for three main reasons:

Firstly, as climatic conditions are the major determinants for the establishment of codling moths, nineteen bioclimatic variables and elevation data were selected to indicate the ecological conditions that are required for codling moth survival and growth, referring to some existing studies^11,19^.

Secondly, the global apple yield data represents the availability of host plant apple trees for codling moths, which is another important indicator used to assess the risk of codling moth establishment. If host plants exist in a region, it might also contain suitable environmental conditions for codling moths.

Thirdly, the long-distance spread of codling moths to new habitats mainly occurs through international trade and transportation. The global accessibility (travel time to major cities) data reflect the connectivity and the concentration of international trade and transportation. As we mentioned before, codling moths have a broad environmental tolerance and are able to opportunistically establish populations in areas with low climate suitability with the assistance of humans; So human factors are indispensable for assessing the potential codling moth distribution.

The three kinds of indicators above comprehensively cover the processes from growth and survival to the spread of codling moth and considering as many aspects as possible, guaranteeing the rationality of the model.

### Maximum entropy model (Maxent)

There are many models used to assess the potential distribution of species. According to some comparative studies on different models, Maxent outperforms GARP^20^ and some presence-only methods (e.g. DOMAIN, ENFA)^21^, have advantages over BIOCLIM^22^. Maxent is a general-purpose machine learning method with a precise mathematical formulation, it has a number of aspects that make it well-suited for species distribution modelling, such as Maxent uses a regularization multiplier to control model complexity and thus avoids over-fitting^20,23,24^, it is possible to analyse the contribution of each environmental variable to the suitability and lower data requirement^25–27^. Therefore, the Maxent niche model has been widely used to model potential species distributions^28–31^. In this study, the Maxent model was selected to simulate the potential risk area of codling moth.

The Maxent model applied a machine learning method called maximum entropy modelling^32^, it follows the principle of maximum entropy: when approximating an unknown probability distribution, the best approach is to ensure that the approximation is subject to any constraints on the unknown distribution^33^. The entropy formula is defined as below:

1 $$H(\hat{\pi })=-\,\sum _{x\in X}\hat{\pi }(x)ln\hat{\pi }(x)$$

where *π* is the unknown probability distribution; $\hat{\pi }$ is the approximation of *π*; *X* is a finite set; *x* is an individual element in set *X*; and *ln* is the natural logarithm. The entropy is nonnegative and is at most the natural log of the number of elements in *X*.

Maxent integrates species presence locations with a set of environmental variables (e.g., temperature, precipitation) across a study area that is divided into grid cells and generates probabilities of species presence or predicted local abundance^20^. Maxent identifies areas that have conditions that are most similar to the current known occurrences of a species and ranks them from 0 (unsuitable) to 1 (most suitable).

## Electronic supplementary material

Supplementary material 1
