# Supplementary material for: Mapping the Potential Global Codling Moth (Cydia pomonella L.) Distribution Based on a Machine Learning Method
Source: Sci Rep. 2018 Aug 30;8:13093. doi: 10.1038/s41598-018-31478-3 (PMC6117298; doi:10.1038/s41598-018-31478-3)
Supplement: Supplementary file 1 — Supplementary material 1 [file 41598_2018_31478_MOESM1_ESM.docx]

**Mapping the Potential Global Codling Moth (*Cydia pomonella L.*) Distribution Based on a Machine Learning Method**

Dong Jiang^1,2,3^ ([jiangd@igsnrr.ac.cn](mailto:jiangd@igsnrr.ac.cn)), Shuai Chen^1,2^ ([chenshuai17@mails.ucas.ac.cn](mailto:chenshuai17@mails.ucas.ac.cn)), Mengmeng Hao ^1,2,*^([haomm.16b@igsnrr.ac.cn](mailto:haomm.16b@igsnrr.ac.cn)), Jingying Fu^1,2^([fujy@igsnrr.ac.cn](mailto:fujy@igsnrr.ac.cn)) & Fangyu Ding^1,2^ ([dingfy.14s@igsnrr.ac.cn](mailto:dingfy.14s@igsnrr.ac.cn))

1 State Key Laboratory of Resources and Environmental Information Systems, Institute of Geographical Sciences and Natural Resources Research, Chinese Academy of Sciences, Beijing 100101, China

2 College of Resource and Environment, University of Chinese Academy of Sciences, Beijing 100049, China

3 Key Laboratory of Carrying Capacity Assessment for Resource and Environment, Ministry of Land &Resources, Beijing 100101, China

Corresponding author: Mengmeng Hao; E-mail: [haomm.16b@igsnrr.ac.cn](mailto:haomm.16b@igsnrr.ac.cn); Tel. +86-10-64889433; Fax: +86-10-64855049.

**Supplementary Figures**


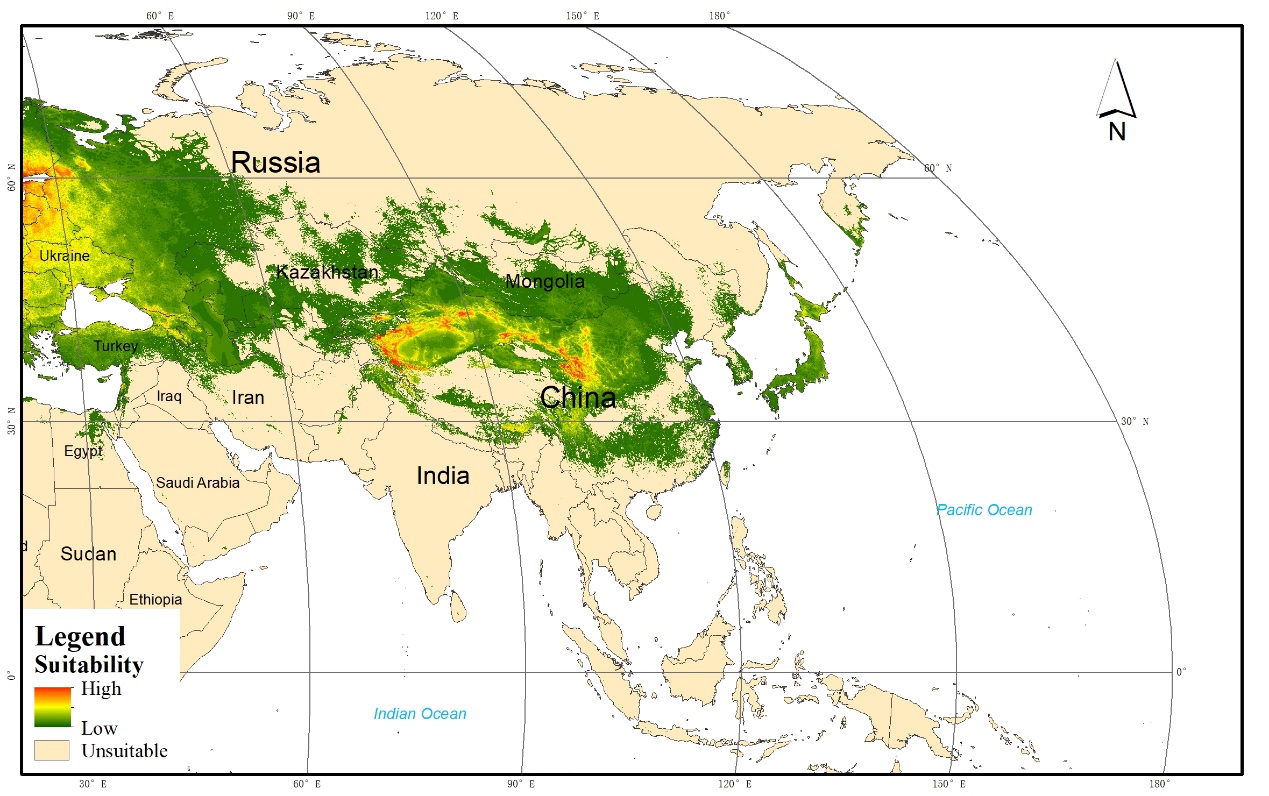


**Figure S1.** Global potential distribution of codling moth in Asia


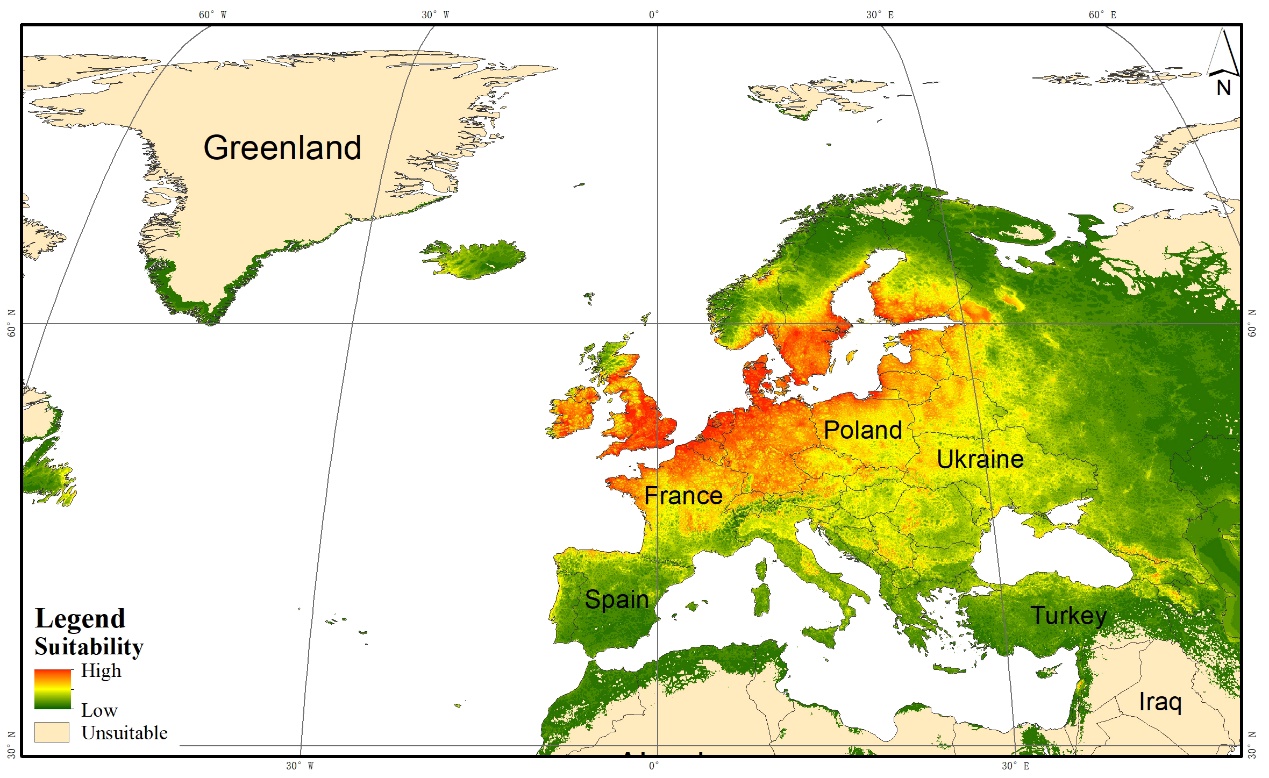


**Figure S2.** Global potential distribution of codling moth in Europe


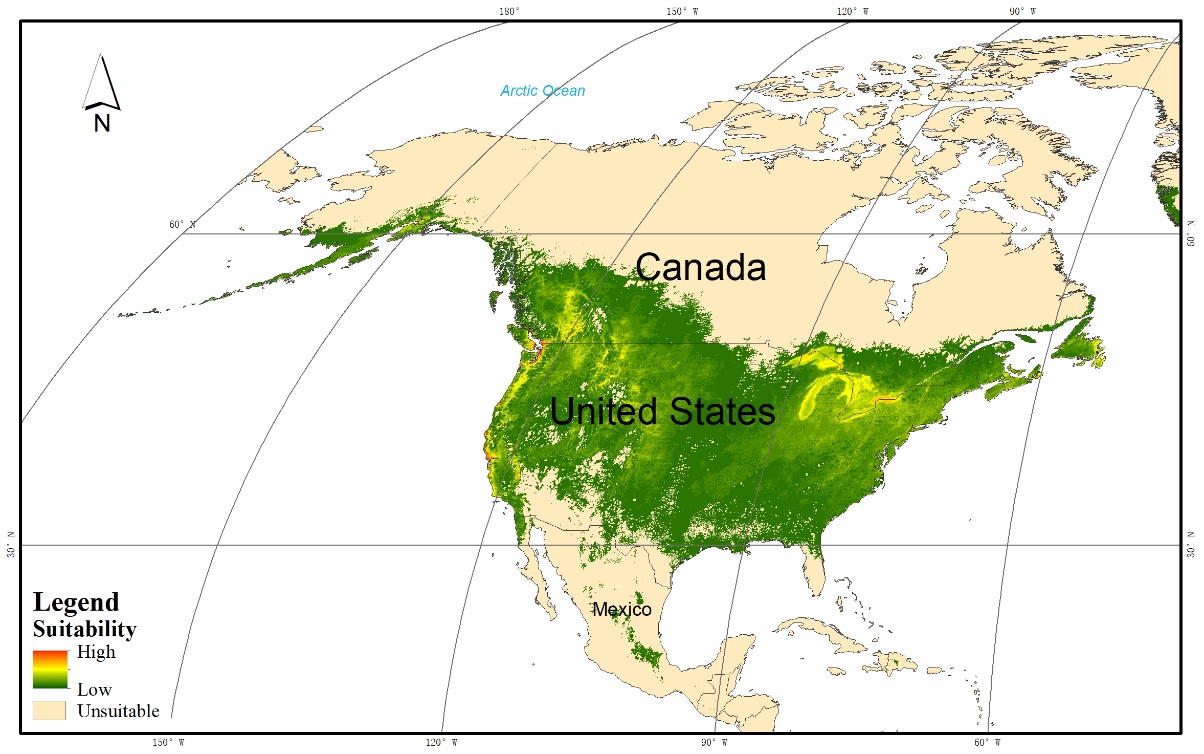


**Figure S3.** Global potential distribution of codling moth in North America


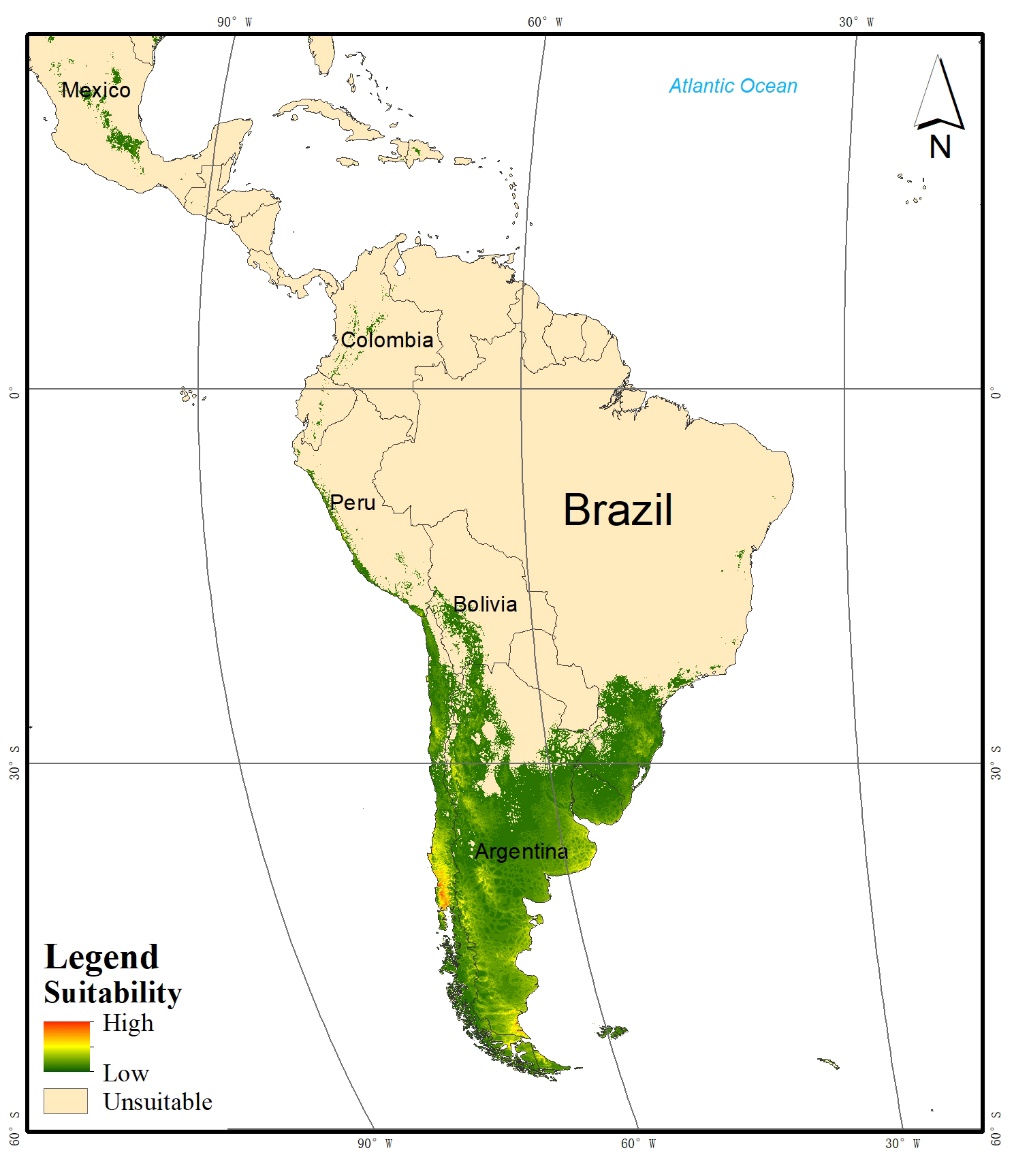


**Figure S4.** Global potential distribution of codling moth in South America


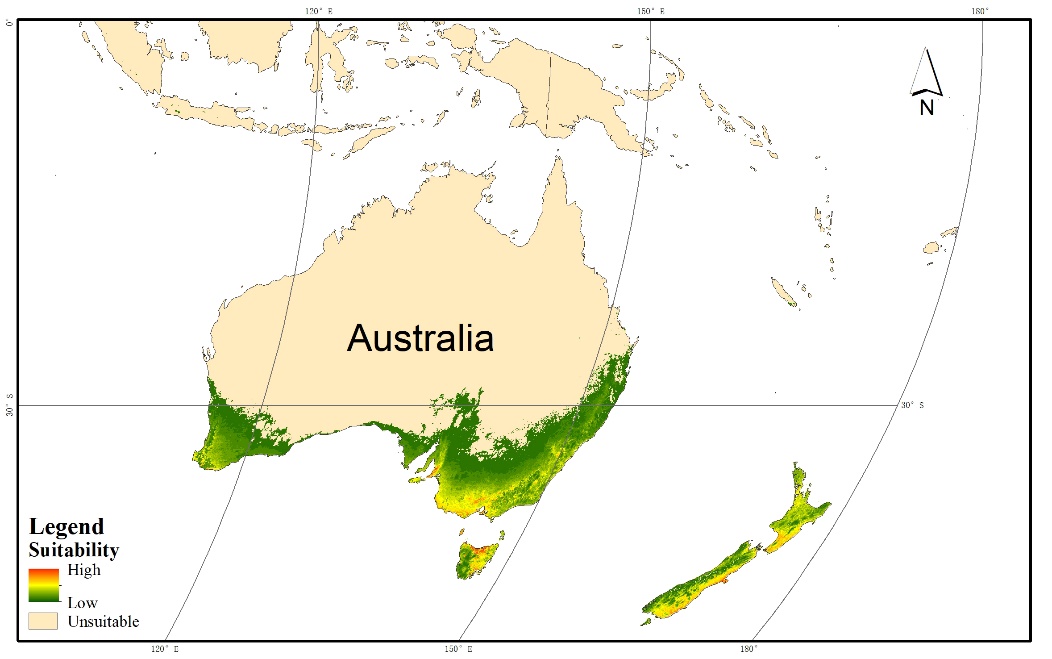


**Figure S5.** Global potential distribution of codling moth in Oceania


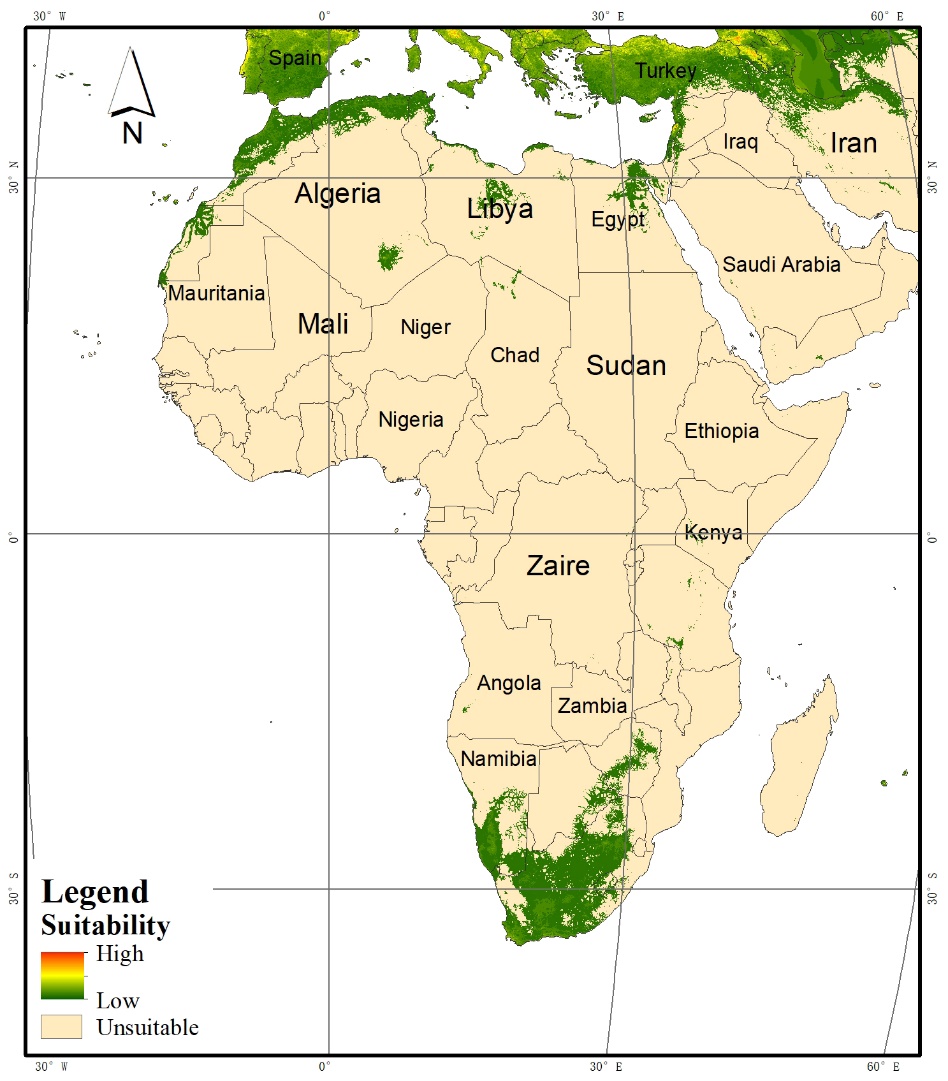


**Figure S6.** Global potential distribution of codling moth in Africa
